# Effect of Body mass index on the performance characteristics of PSA-related markers to detect prostate cancer

**DOI:** 10.1038/srep19034

**Published:** 2016-01-12

**Authors:** Yao Zhu, Cheng-Tao Han, Gui-Ming Zhang, Fang Liu, Qiang Ding, Jian-Feng Xu, Adriana C. Vidal, Stephen J. Freedland, Chi-Fai Ng, Ding-Wei Ye

**Affiliations:** 1 Department of Urology, Fudan University Shanghai Cancer Center, Shanghai, P.R. China; 2 Department of Oncology, Shanghai Medical College, Fudan University, Shanghai, P.R. China; 3 State Key Laboratory of Genetic Engineering, School of Life Sciences, Fudan University, Shanghai, P.R. China; 4 Fudan Institute of Urology, Huashan Hospital, Fudan University, Shanghai, P.R. China; 5 Department of Urology, Huashan Hospital, Fudan University, Shanghai, P.R. China; 6 Center for Genetic Epidemiology, School of Life Sciences, Fudan University, Shanghai, P.R. China; 7 Department of Surgery, Center for Integrated Research on Cancer and Lifestyle, Samuel Oschin Comprehensive Cancer Institute, Cedars Sinai Medical Center, Los Angeles, CA; 8 SH Ho Urology Centre, Department of Surgery, The Chinese University of Hong Kong, Shatin, NT, Hong Kong SAR, P.R. China

## Abstract

To examine whether the predictive performance of prostate-specific antigen (PSA) and PSA-related markers for prostate cancer (PCa) is modified by body mass index (BMI). Patients with a PSA 2–10 ng/mL who underwent multicore prostate biopsies were recruited from three tertiary centers. Serum markers measured included total PSA (tPSA), free-to-total PSA (f/tPSA), p2PSA, percentage of p2PSA (%p2PSA), and prostate health index (PHI). The association between serum markers and PCa risk was assessed by logistic regression. Predictive performance for each marker was quantified using the area under the receiver operator curves (AUC). Among 516 men, 18.2% had PCa at biopsy. For all tested markers, their predictive value on PCa risk was lower in obese patients compared to normal weight patients. We found statistically significant interactions between BMI and tPSA (*P* = 0.0026) and p2PSA (*P* = 0.038). PHI achieved an AUC of 0.872 in normal weight patients and 0.745 in obese patients, which outperformed the other predictors regardless of BMI category. In conclusion, PHI achieved the best predictive performance for detecting PCa and was not influenced by BMI.

Over the last decade, a steady increase in prostate cancer (PCa) incidence has been observed in the People’s Republic of China^1^. In developed areas such as Shanghai, an annual increase of 8% was reported and PCa was ranked as the fifth most common male malignancy in this region after 2010^1^. The emerging incidence of PCa may be partly explained by the widespread use of prostate-specific antigen (PSA) testing in urban areas. We previously examined patient characteristics in patients undergoing radical prostatectomy between 2012 and 2014, and found that 64.3% of those patients had increased PSA levels but no PCa symptoms, suggesting wide-spread PSA screening^2^. In contrast, in 2005 the percentage of patients undergoing radical prostatectomy with T1c disease (elevated PSA only) was as low as 10%, as reported by a multicenter study from the People’s Republic of China^3^.

Over the past 15 years, not only has PCa incidence increased, but similar trends have been noted in increased body mass index rates^4^. Hou *et al.* compared two cross-sectional surveys (1998–2001 vs. 2007–2008) that investigated the prevalence of obesity among Chinese adults in urban Shanghai^5^, and found that among men during that period, the prevalence of obese (body mass index [BMI] ≥ 25) increased from 31.5% to 39.1% while metabolically active obesity (i.e. central obesity) increased from 19.5% to 27.3%.^5^

The difficulty of detecting PCa in obese men is well recognized^6^. One of the main reasons cancers can be harded to find in obese men is that obese subjects have lower serum PSA values due to hemodilution^7^. However, detection biases of serum markers could be minimized or even reversed by the higher probability of PCa in obese individuals^8^. Recently, Abrate *et al.* assessed the effect of obesity on the predictive value of PSA-related markers in European patients^9^. Their findings indicated that PSA and all PSA-based markers (p2PSA, %p2PSA, and PHI) except free PSA and percent free PSA performed better in obese men. However, in general there were no differences between normal weight and obese subjects arguing against a dose-response effect. As such, there is no clear logical explanation for those findings. Indeed, multiple other studies suggest that obesity does not modify the ability of PSA in predicting PCa^10^,^11^. To what degree those results can be generalized to Chinese men is unclear because there is a pronounced difference in PCa incidence between Chinese and Caucasian populations. For men with PSA 4–10 ng/mL, the detection rate of PCa may in Caucasian men may be up to 40%^9^,^11^, but only approximately 20% in Chinese men^12^. Also, how BMI affects other serum based markers beyond PSA has not been tested to date with the exception of the study by Abrate *et al.*

Herein, we examined the associations between PSA-related markers and PCa risk according to BMI categories among men undergoing prostate biopsy. Specifically, we tested for interactions between BMI and serum makers to assess whether BMI affected the ability of these PSA-based markers to predict PCa diagnosis. Furthermore, we used head-to-head comparisons to find predictors with superior discriminative ability in different BMI subgroups. We hypothesized that the predictive value of PSA serum markers to detect PCa at biopsy would not be influenced by BMI.

## Results

### Baseline study characteristics

Of 516 men included in this study, PCa was diagnosed in 94 (18.2%) subjects, including 64 (12.4%) with Gleason score ≥7. Using a BMI cutoff of 25 kg/m^2^ 13, 188 (36.4%) men were defined as being obese (BMI ≥ 25 kg/m^2^) at the time of biopsy. There was no significant difference in demographic or clinical characteristics between normal weight and obese men (all p ≥ 0.068) (Table 1). Similarly, normal weight and obese men had similar rates of PCa detection including similar risks of high-grade disease (all ≥ 0.594).

### Effect of serum based markers on PCa risk stratified by BMI

As shown in Table 2, logistic regression analysis models revealed the effect size (odds ratios, OR) of serum predictors on PCa risk in the entire sample and stratified by BMI. Although tPSA was significantly associated with PCa risk in the normal weight subgroup (OR = 1.369, p < 0.0001), the association was lost in the obese subgroup (OR = 0.962, p = 0.661) (p-interaction = 0.0026). A similar trend was also observed for p2PSA, and its effect size was halved in obese men (OR = 1.063, p = 0.038) compared with their normal weight counterparts (OR = 1.132, p < 0.001) (p-interaction = 0.038). While a similar trend was noticed for PHI (i.e. stronger in normal weight men compared to obese men), it was a strong predictor in both groups (both p < 0.001) and the interaction was not statistically significant (p-interaction = 0.085).

We further evaluated which serum marker had the best discriminative ability among normal weight and obese subgroups, respectively. The AUC of all predictors was lower in the obese samples (Table 3). Among normal weight men, PHI had the highest discriminative ability among the five predictors examined with an AUC of 0.872 and a specificity of 60.9% when sensitivity was set to 90%. Indeed, its accuracy was significantly greater than all markers (all p ≤ 0.001). In the obese group, PHI still achieved the highest AUC of 0.745 and remained significantly better than all other markers (all p≤0.01) except %p2PSA, which had similar accuracy (AUC 0.731).

### Effect of serum based markers on high-grade PCa risk stratified by BMI

Finally, we evaluated the influence of BMI on serum markers’ effect size (Supplementary Table 1) and discriminative ability (Supplementary Table 2) for detecting high-grade disease. Importantly, all markers except tPSA (p-interaction = 0.014) showed equal ability to detect high-grade disease in normal weight and obese men (all p-interaction ≥ 0.59). Similar to predicting all cancers, PHI had the best discriminative accuracy in normal weight men (AUC 0.885) and obese men (AUC 0.839). Also, PHI outperformed all other markers in normal weight men (all p ≤ 0.001) and all other markers in obese men (all p ≤ 0.025) except %p2PSA (p = 0.806).

## Discussion

In this multicenter study of men who underwent extended biopsy, we tested whether the predictive performance of serum PSA-related tumor markers for PCa was influenced by obesity. Our findings showed a weaker association with PCa in obese men for all tested markers. The interaction between obesity and serum predictors was statistically significant for tPSA and p2PSA, but not for PHI. PHI achieved better discriminative ability for PCa than PSA and its derivatives in both normal weight and obese men.

There are several possible ways by which obesity may influence the predictive performance of serum markers^6^. First, the hemodilution effect of PSA is well recognized in obese men^7^. The implication is that circulating PSA levels released from PCa tissue are diluted in obese men due to greater blood volume leading to lower PSA levels. However, in our study, we did not see lower tPSA levels in obese men. In fact, obese men had slightly higher tPSA levels, though this was not statistically significant. The hemodilution theory, as PSA is simply diluted and the association with PCa is not fundamentally changed, it is anticipated that this would have no effect on BMI modifying the ability of serum markers to detect PCa.

An alternative way that obesity may affect the performance of serum markers is that obese men have larger prostate volumes. This poses a problem from two aspects. First, this increases the probability of false-negative biopsies (i.e. difficulty finding the cancer). Second, larger prostates result in larger PSA values, independent of PCa, thereby decreasing the accuracy of PSA. Indeed, treatment for enlarged prostate with a 5-alpha reductase inhibitor, which shrinks the prostate, results in improved accuracy of PSA for predicting total and high-grade prostate cancer^14^. However, while prostate volumes in our cohort were larger in the obese men, the differences compared to normal weight men were small and not significant. Nonetheless, larger prostates and hence greater degree of benign prostatic hyperplasia in obese men would be expected to worsen serum markers ability to detect PCa in obese men.

A third way that obesity may influence serum-markers is that obesity may be related to different subtypes of PCa^15^ differently. Specifically, in PSA-screened men, obesity appears to be associated with low risk of low-grade PCa and higher risk of high-grade PCa^6^. As most serum markers perform better for high-grade disease, this would be expected to result in serum markers performing better in obese men.

In summary, depending on the potential mechanism, serum markers could be postulated to work similarly, worse, or better in obese men. Given this controversy, it is of note that multiple prior studies have addressed the performance of serum markers for detecting PCa as a function of elevated BMI (Supplementary Table 3)^9^,^11^,^16^,^17^,^18^,^19^. Among these seven studies (including the current study), three reported notable alterations in PSA performance by BMI categories whereas four, including two from Korea, showed no differences. Among the three studies that found obesity altered PSA’s performance, Abrate *et al.* found an increased performance of PSA among obese European men^9^. Conversely, our group and Chiu *et al.*^16^ showed the reverse relationship in Chinese men. The reasons for these discrepancies are not clear. However, we do note that the cancer detection rate in the two Chinese studies (16–18%) is much lower than the other studies (22–43%). Also, the two Chinese studies had the lowest percentage of men with “elevated” BMI (defined as >25 kg/m^2^ in the current study). Thus, it remains plausible that geographic differences and underlying PCa biological differences across ethnic groups could contribute to our findings. However, our findings are consistent with the only prior study from China, lending some credibility to our findings. Future research is needed to better outstand how geographic and ethnic differences in PCa may alter the ability to detect PCa as a function of obesity and current serum based markers.

Given the high rate of negative biopsies (>80% in the current study), despite an elevated PSA of 2–10 ng/ml, new and better serum markers are needed. Within our cohort, PHI was the strongest predictor of overall and high-grade PCa and importantly its performance was not influenced by BMI. Other studies have also shown that PHI is an accurate predictor of PCa including in Chinese patients^20^,^21^. As such, future studies should further explore PHI as a strong predictor of PCa, including high-grade disease.

The strength of our study is its multicenter design, head-to-head comparisons of multiple serum markers, and that we enrolled a contemporary cohort of prostate biopsies in an under-reported population. However, the current study has several limitations. First, patients were recruited consecutively from three tertiary centers in Shanghai and Hong Kong. Thus, our results should be interpreted with caution for possible selection bias and may not be applicable to community-based series. Second, although all centers were experienced in multicore prostate biopsies and pathological examination, the possibility of underdiagnoses should be acknowledged. Third, all men were selected for biopsy due to an elevated PSA and those with very high PSA values (>10 ng/ml) were excluded. This creates a narrow and truncated range for PSA and thus inhibits its ability to predict PCa. Moreover, it results in us being unable to assess the performance of the other markers in a broad secreening population. Finally, the drawbacks of BMI for characterizing adiposity have been recognized^22^. Therefore, our study should be considered as one step toward optimizing tumor marker use in the context of an obesity epidemic. We are prospectively collecting more data, such as waist circumference and waist-to-hip ratio, for further analyses.

In conclusion, in our cohort of men undergoing prostate biopsy in China with a cancer detection rate of 18%, all serum markers, except PHI, performed better in normal weight men relative to obese men. Overall, PHI had the best performance characteristics for detecting overall and high-grade PCa and was not influenced by BMI. PHI appears to be a promising biomarker for predicting overall and high-grade PCa among men in the PSA gray zone of 2-10 ng/ml.

## Material and Methods

### Study population

Patients were recruited from three tertiary centers, two from Shanghai (Fudan University Shanghai Cancer Center and Fudan University Huashan Hospital) and one from Hong Kong (Chinese University of Hong Kong). The period for recruitment was between April 2012 and August 2014 for Shanghai and between April 2008 and April 2013 for Hong Kong. Biopsy indication was PSA >4 ng/mL or abnormal digital rectal examination (DRE). Patients underwent transrectal ultrasound-guided prostate biopsies according to a standardized extended scheme with at least 10 cores. Specific genitourinary pathologists evaluated the biopsy tissue. Serum taken prior to biopsy was assayed for [-2]proPSA (p2PSA, pg/ml), total PSA (tPSA, ng/ml), and free PSA (fPSA, ng/ml) using Beckman Coulter’s DxI 800 Immunoassay system in a central laboratory. f/tPSA and %p2PSA were calculated as 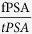 and 
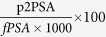
, respectively. Prostate health index (PHI) was calculated according to the following formula: 
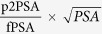
. BMI was categorized as normal weight (<25 kg/m^2^) vs. obese (≥25 kg/m^2^) according to World Health Organization and National Institute of Health classifications^23^. Few patients had a BMI ≥ 30 kg/m^2^ (n = 12).

For the current analyses, the inclusion criteria were an initial prostate biopsy and PSA of 2–10 ng/mL. We excluded patients with acute prostate infection (National Institute of Health Classification, Category I^24^), history of 5-α reductase inhibitor use within 6 months prior to biopsy, and a serum sample archived for more than 3 years. This study was carried out in accordance with the ethical standards of Helsinki Declaration II and approved by the Institution Review Boards of Fudan University Shanghai Cancer Center, Fudan University Huashan Hospital, and Chinese University of Hong Kong. Written informed consent was obtained from each patient before any study-specific investigation was performed.

### Statistical Analysis

Our primary outcome was PCa diagnosis at biopsy by BMI category and our exposures were serum total PSA, free PSA and [–2] proPSA (p2PSA). Free-to-tPSA ratio (%fPSA), p2PSA-to-tPSA ratio, PSA density (PSAD), and PHI, were calculated, using standard formulas. Continuous variables are presented as median and interquartile ranges, and categorical variables are presented as nominal numbers (or numerical values) and percentages. For comparisons, the Chi-square and rank sum tests were used for categorical variables and continuous variables, respectively. Logistic regression models were used to test the associations between serum markers and PCa risk at biopsy stratified by BMI category. As markers were collinear, separate models were run for each marker. Interaction terms between serum markers and BMI categories for predicting PCa were then assessed by using a cross-product term in the respective model. Discriminative ability was quantified as area under the receiver operating characteristic curve (AUC). The DeLong method was used to compare AUCs. All statistical analyses were performed using R and publicly available packages. Significance was set at *P* < 0.05.

## Additional Information

**How to cite this article**: Zhu, Y. *et al.* Effect of Body mass index on the performance characteristics of PSA-related markers to detect prostate cancer. *Sci. Rep.* **6**, 19034; doi: 10.1038/srep19034 (2016).

## Supplementary Material

Supplementary Information

## Figures and Tables

**Table 1:** Characteristics of the study sample stratified by BMI.

Variable	Stratified by BMI				*P*	
	Normal weight (<25)		Overweight ( ≥ 25)			
n	328		188			
Site (%)					0.184	
1	230	(70.1)	121	(64.4)		
2	36	(11)	31	(16.5)		
3	62	(18.9)	36	(19.1)		
Biopsy cores (median [IQR])	12	[11.5, 12]	12	[12, 12]	0.85	
Age, year (median [IQR])	65	[59.75, 72.00]	64	[59.00, 70.00]	0.165	
BMI, kg/m^2^ (median [IQR])	22.59	[21.11, 23.88]	26.54	[25.69, 27.61]	<0.001	
DRE abnormal (%)	49	(16.2)	22	(12.5)	0.339	
Prostate volume, ml (median [IQR])	40	[29.25, 54.00]	42	[33.25, 55.00]	0.068	
tPSA, ng/ml (median [IQR])	6.62	[5.00, 8.00]	7.00	[5.10, 8.33]	0.185	
f/tPSA (median [IQR])	0.18	[0.13, 0.25]	0.17	[0.12, 0.22]	0.239	
p2PSA, pg/ml (median [IQR])	12.95	[8.98, 19.23]	12.96	[9.55, 19.00]	0.89	
PHI (median [IQR])	31.09	[22.47, 43.32]	33.84	[24.97, 42.70]	0.285	
%p2PSA (median [IQR])	1.22	[0.90, 1.71]	1.24	[0.91, 1.61]	0.655	
Biopsy outcome = cancer (%)	57	(17.4)	37	(19.7)	0.594	
Gleason score ≥7 (%)	43	(13.1)	21	(11.2)	0.614	

Abbreviations: BMI = body mass index, DRE = digital rectal examination, PSA = prostate-specific antigen, PHI =  prostate health index, IQR = interquartile range.

**Table 2:** Associations of predictors with prostate cancer risk in the entire sample and stratified by BMI.

Predictor	Whole sample		Normal weight		Obese		
	OR	*P*	OR	*P*	OR	*P*	*P*_*interaction*_
tPSA	1.196	0.0015	1.369	<0.0001	0.962	0.6612	0.0026
f/tPSA	0.004	0.0002	0.004	0.0023	0.006	0.0372	0.855
p2PSA	1.102	<0.0001	1.132	<0.0001	1.063	0.0071	0.038
PHI	1.092	<0.0001	1.106	<0.0001	1.071	<0.0001	0.085
%p2PSA	4.559	<0.0001	4.807	<0.0001	4.293	<0.0001	0.789

Statistics were calculated using logistic regression models.

Abbreviations: BMI = body mass index, OR = odds ratios, PSA = prostate-specific antigen, PHI = prostate health index.

**Table 3:** Receiver operating characteristic curve analyses of predictors for prostate cancer risk stratified by BMI.

Predictor	Normal weight					Overweight				
	AUC	95% C.I.	*P*-test with PHI	Specificity at 90% sensitivity	95% C.I.	AUC	95% C.I.	*P*-test with PHI	Specificity at 90% sensitivity	95% C.I.
tPSA	0.675	0.609-0.741	<0.0001	0.398	0.300-0.476	0.527	0.424-0.630	0.0025	0.205	0.024–0.285
f/tPSA	0.646	0.567–0.725	<0.0001	0.135	0.077–0.358	0.595	0.500–0.690	0.0068	0.199	0.106–0.437
p2PSA	0.758	0.690–0.825	0.0014	0.343	0.244–0.541	0.618	0.514–0.722	0.01	0.185	0.073–0.371
PHI	0.872	0.816–0.927	reference	0.609	0.343–0.819	0.745	0.642–0.847	reference	0.205	0.026–0.587
%p2PSA	0.769	0.702–0.835	<0.0001	0.423	0.234–0.615	0.731	0.624–0.838	0.841	0.171	0.027–0.637

Abbreviations: BMI = body mass index, AUC = area under the curve, C.I. = confidence interval, PSA = prostate-specific antigen, PHI = prostate health index.
