# Supplementary material for: Effect of Body mass index on the performance characteristics of PSA-related markers to detect prostate cancer
Source: Sci Rep. 2016 Jan 12;6:19034. doi: 10.1038/srep19034 (PMC4709513; doi:10.1038/srep19034)
Supplement: Supplementary Information [file srep19034-s1.pdf]

# Effect of Body mass index on the performance characteristics of PSA-related markers to detect prostate cancer

Yao Zhu[¶1,2], Cheng-Tao Han[¶1,2], Gui-Ming Zhang[1,2], Fang Liu[3], Qiang Ding[4,5], Jian-Feng Xu[3,4,5,6], Adriana C. Vidal[7], Stephen J. Freedland[7], Chi-Fai Ng[*8], Ding-Wei Ye[*1,2]

[¶]Co-first authors

**Supplementary Table 1.** Associations of predictors with high-grade cancer risk in the entire sample and stratified by BMI.

| Predictor | Whole sample | | Normal weight | | Overweight | | $P_{interaction}$ |
|---|---|---|---|---|---|---|---|
| | OR | *P* | OR | *P* | OR | *P* | |
| tPSA | 1.230 | 0.0023 | 1.385 | 0.0002 | 0.977 | 0.84 | 0.014 |
| f/tPSA | 0.001 | 0.0001 | 0.001 | 0.0008 | 0.003 | 0.054 | 0.75 |
| p2PSA | 1.114 | <0.0001 | 1.120 | <0.0001 | 1.106 | 0.0003 | 0.73 |
| PHI | 1.099 | <0.0001 | 1.100 | <0.0001 | 1.096 | <0.0001 | 0.9 |
| %p2PSA | 5.842 | <0.0001 | 5.296 | <0.0001 | 6.903 | <0.0001 | 0.59 |

Statistics were calculated using a logistic regression model.
Abbreviations: OR=odds ratios, PSA=prostate-specific antigen, PHI=prostate health index.

**Supplementary Table 2.** Receiver operating characteristic curve analyses of predictors for high-grade cancer stratified by BMI.

| Predictor | Normal weight | | | | | Overweight | | | | |
|---|---|---|---|---|---|---|---|---|---|---|
| | AUC | 95% C.I. | *P* test with PHI | Specificity at 90% sensitivity | 95% C.I. | AUC | 95% C.I. | *P* test with PHI | Specificity at 90% sensitivity | 95% C.I. |
| tPSA | 0.682 | 0.608–0.756 | <0.0001 | 0.384 | 0.284–0.478 | 0.519 | 0.382–0.656 | 0.0002 | 0.216 | 0.010–0.359 |
| f/tPSA | 0.676 | 0.591–0.761 | <0.0001 | 0.229 | 0.091–0.495 | 0.615 | 0.502–0.728 | 0.0008 | 0.367 | 0.177–0.457 |
| p2PSA | 0.748 | 0.674–0.823 | 0.0006 | 0.356 | 0.266–0.615 | 0.708 | 0.590–0.827 | 0.025 | 0.332 | 0.215–0.593 |
| PHI | 0.885 | 0.826–0.945 | reference | 0.768 | 0.484–0.874 | 0.839 | 0.735–0.942 | reference | 0.533 | 0.180–0.862 |
| %p2PSA | 0.791 | 0.720–0.863 | <0.0001 | 0.451 | 0.120–0.733 | 0.821 | 0.711–0.932 | 0.806 | 0.348 | 0.156–0.796 |

Abbreviations: AUC=area under the curve, C.I.=confidence interval, PSA=prostate-specific antigen, PHI=prostate health index.

**Supplementary Table 3**. Literature review of PSA performance for prostate cancer according to BMI stratification.

| First author | Study time | Country | n | Percentage of cancer | PSA (mean) | Prostate volume (mean) | Age (mean) | Cutoff of BMI | Percentage of BMI distribution | AUC performance of PSA (normal weight vs. overweight or ascending, *p* test) |
|---|---|---|---|---|---|---|---|---|---|---|
| Kim JH | 2009–2013 | Korea | 1062 | 21.7 | 6.05 | 44.1 | 67.1 | 25 | 60.9/39.1 | 0.584 vs. 0.633, *P* = 0.234 |
| Banez L | 2002–2010 | Italy | 917 | 43.2 | 6.7# | 47# | 68# | 25/30 | 29.6/49.4/21.0 | 0.61 vs. 0.62 vs. 0.63, *P* = 0.85 |
| Oh JJ | 2004–2011 | Korea | 3471 | 31.7 | 6.0# | 46.1 | 64.1 | 23/25/30 | 28.5/32.4/37.4/1.7 | 0.638 vs. 0.572 vs. 0.613 vs. 0.544, *P* > 0.05 |
| Abrate A | 2011–2012 | Europe | 965 | 43 | 6.8 | 52# | 65.1 | 25/30 | 39.7/45.6/14.7 | 0.566 vs. 0.536 vs. 0.694, *P* < 0.02 |
| Chiu PK | 2009–2012 | China | 854 | 15.6 | 8.2/7.9* | n.m. | 65.9 | 27 | 81.1/18.9 | 0.623 vs. 0.507, n.m. |
| Current study | 2008–2014 | China | 516 | 18.2 | 6.9 | 40.6 | 65 | 25 | 63.6/36.4 | 0.675 vs. 0.527, *P* = 0.0026 |

# median; * 8.2 for normal weight and 7.9 for overweight.

Abbreviations: PSA=prostate specific antigen, BMI=body mass index, AUC=area under the curve, n.m.=not mentioned.
